# Incremental prognostic value of the T-helper 1/T-helper 2 immune phenotype and 72-h change in sequential organ failure assessment for in-hospital mortality in sepsis with and without type 2 diabetes: a retrospective two-cohort study

**DOI:** 10.3389/fmed.2026.1911557

**Published:** 2026-10-12

**Authors:** Dongxu Pan, Zongnan Lv, Jiayan Li, Bo Zhang, Feifei Xu, Wuxiang Huang, Wei Chen, Xuejun Mo

**Affiliations:** 1 Department of Intensive Care Medicine, Minzu Hospital of Guangxi Zhuang Autonomous Region/Affiliated Minzu Hospital of Guangxi Medical University, Nanning, Guangxi, China; 2 Department of Endocrinology and Metabolism, Minzu Hospital of Guangxi Zhuang Autonomous Region/Affiliated Minzu Hospital of Guangxi Medical University, Nanning, Guangxi, China

**Keywords:** immunometabolism, interleukin-6, mortality prediction, sepsis, sequential organ failure assessment, Th1/Th2 ratio, type 2 diabetes mellitus, ΔSOFA

## Abstract

**Background:**

Type 2 diabetes mellitus (T2DM) is accompanied by chronic immunometabolic dysregulation and may alter the host response to sepsis. We compared immunometabolic profiles and early organ dysfunction trajectories in patients with sepsis with and without T2DM and evaluated 72-h mortality risk reassessment models.

**Methods:**

Of 1,200 consecutive adult intensive care unit (ICU) patients screened between January 2020 and December 2025, 1,023 met Sepsis-3 criteria and constituted the 72-h landmark cohort, defined by an ICU stay of at least 72 h and paired day-1/day-3 Sequential Organ Failure Assessment (SOFA) measurements; all model comparisons were therefore conditional on survival and evaluability at 72 h. Patients were classified into a T2DM-sepsis cohort (*n* = 513) or non-T2DM-sepsis cohort (*n* = 510). Model 1 included day-1 SOFA; Model 2 added the T-helper 1/T-helper 2 (Th1/Th2) ratio and interleukin-6 (IL-6); and Model 3 further added the 72-h change in SOFA (ΔSOFA; day-3 minus day-1 SOFA). *Post hoc* secondary analyses evaluated age-only and age-adjusted benchmarks and a pooled model containing diabetes status, age, and interaction terms. Internal validation was performed using 1,000 bootstrap resamples.

**Results:**

Compared with patients without T2DM, those with T2DM had higher glycemic, inflammatory, renal, and physiological severity measures. In the T2DM cohort, the apparent area under the receiver operating characteristic curve (AUC) was 0.781 for Model 1, 0.838 for Model 2, and 0.861 for Model 3; the optimism-corrected Model 3 AUC was 0.858. Age alone had an AUC of 0.634, whereas adding age to Model 3 yielded an AUC of 0.870 (DeLong *P* = 0.099 vs. Model 3). The age-adjusted pooled model had an apparent AUC of 0.862, and the global predictor-by-diabetes interaction test was not statistically significant (*P* = 0.191).

**Conclusion:**

A 72-h reassessment model combining baseline organ dysfunction, early SOFA trajectory, the Th1/Th2 ratio, and IL-6 may improve mortality risk stratification beyond SOFA alone. The model is intended to prioritize structured clinical reassessment, not replace SOFA or prescribe treatment. Temporal and multicenter, external validation is required before clinical translation.

## Introduction

Sepsis is life-threatening organ dysfunction caused by a dysregulated host response to infection and remains a major cause of death in intensive care medicine (1, 2). The 2021 Surviving Sepsis Campaign guidelines stress the importance of timely antimicrobial therapy and organ support, as well as repeated assessment of severity, response to treatment, and prognosis (3). Sepsis, however, is not a uniform clinical entity. Patients with similar pathogens and comparable Sequential Organ Failure Assessment (SOFA) scores may differ substantially in immune status, metabolic background, and organ reserve. Identifying high-risk phenotypes within this broad diagnostic category remains a central challenge for precision critical care.

Type 2 diabetes mellitus (T2DM) adds a distinct layer of biological and clinical complexity. Chronic hyperglycemia, lipotoxicity, insulin resistance, endothelial dysfunction, and low-grade inflammation are common in T2DM and may influence both susceptibility to infection and the host response to severe infection (4). Systematic evidence indicates that the association between diabetes and sepsis outcomes is complex rather than consistently harmful, and that it may vary according to the sepsis definition, infection site, baseline organ function, glycemic management, and immune phenotype (5). Sepsis in patients with T2DM should therefore be considered an acute infectious syndrome occurring against the background of a preexisting immunometabolic disorder, rather than simply “sepsis plus a chronic disease.”

We therefore analyzed the T2DM and non-T2DM cohorts in parallel to determine whether immune-inflammatory markers and early SOFA change showed similar directions and incremental prognostic value in two distinct immunometabolic settings. We did not assume that cohort-specific models would necessarily outperform a pooled model. Accordingly, we conducted *post hoc* sensitivity analyses using a pooled model that included diabetes status and age and formally tested predictor-by-diabetes interactions. Because these analyses were not part of the original model-development plan, they are reported as secondary analyses.

T-helper cell polarization is a biologically integral component of the immune response in sepsis. T helper 1 (Th1) cells primarily support cellular immunity, macrophage activation, and pathogen clearance, whereas T helper 2 (Th2) cells contribute to humoral immunity and anti-inflammatory regulation. Current immune profiling studies suggest that sepsis does not progress in a linear trajectory from hyperinflammation to immunosuppression. Instead, inflammatory activation, immunosuppression, endothelial injury, coagulopathy, and metabolic reprogramming coexist and interact (6). The Th1/Th2 ratio may therefore provide a practical summary of T-cell-mediated immune polarization, especially when considered alongside cytokines such as interleukin-6 (IL-6) and interleukin-10 (IL-10).

The SOFA score is central to Sepsis-3 diagnosis and prognostic assessment, and it should be interpreted within its theoretical range of 0–24 points (3). In practice, changes in SOFA scores after initial treatment may be as informative as the baseline value. The 72-h change in SOFA (ΔSOFA), calculated as the day-3 SOFA minus the day-1 SOFA, reflects early organ dysfunction trajectories. A falling score suggests clinical resolution, whereas a rising score may indicate persistent inflammation, impaired perfusion, microcirculatory dysfunction, or ongoing immunometabolic collapse. Recent evidence also supports the prognostic value of such dynamic, trajectory-based SOFA indices in sepsis (7).

In this study, we compared baseline characteristics, cytokine profiles, the Th1/Th2 immune phenotype, and early SOFA trajectories between patients with sepsis with and without T2DM. We then evaluated three stepwise mortality models: Model 1 incorporated day-1 SOFA alone; Model 2 added admission Th1/Th2 and IL-6; and Model 3 further added ΔSOFA. The unmet clinical need is not another general sepsis severity score. Rather, clinicians need a structured framework to distinguish, at the 72-h reassessment milestone, patients who exhibit similar baseline severity and SOFA trajectories but different immunometabolic risk profiles. The proposed model is intended as a secondary prioritization aid alongside SOFA, not as a replacement for it. Operationally, it may help identify patients who warrant a multidisciplinary review of source control, diagnostic imaging, antimicrobial adequacy, hemodynamic perfusion targets, evolving organ support needs, monitoring intensity, and, when appropriate, prognosis and goals of care. The model does not direct a specific intervention, and this retrospective study cannot establish that model-guided care improves outcomes.

## Materials and methods

### Study design and participants

This single-center retrospective two-cohort, observational study was conducted in the intensive care unit (ICU) of our hospital. A total of 1,200 consecutive adult patients admitted between January 2020 and December 2025 were screened. Sepsis was defined according to Sepsis-3 criteria: suspected or confirmed infection accompanied by an acute increase in SOFA score of at least 2 points (1, 3). The study is reported in accordance with the STROBE guidelines statement and relevant TRIPOD+AI recommendations for clinical prediction models developed using multivariable regression (8).

Patients were eligible if they were at least 18 years old, met Sepsis-3 criteria, remained in the ICU for at least 72 h, had paired observed SOFA scores on ICU days 1 and 3, and had key clinical and immunological assessments available within 24 h after ICU admission. These criteria defined the 72-h landmark cohort. Model 3 was designed for risk reassessment on day 3. Because Models 1–3 were evaluated in the same landmark cohort, every performance comparison was conditional on survival and data availability at 72 h. Results for Models 1 and 2 therefore should not be interpreted as admission-day performance among all consecutive patients with sepsis. For recurrent ICU admissions, only the index episode was included. T2DM status was determined from medical records, discharge diagnoses, antidiabetic medication records, and available HbA1c or blood glucose measurements, using established clinical criteria consistent with the American Diabetes Association Standards of Care (9).

Of the 1,200 screened patients, 177 met at least one predefined exclusion criterion: premature discontinuation or incomplete evaluation related to adverse drug reactions; malignant neoplasms, hematological diseases, or autoimmune diseases; long-term use of immunosuppressive agents or systemic glucocorticoids; pregnancy or lactation; missing key clinical, outcome, or immunological data; or an ICU stay shorter than 72 h, a missing day-3 SOFA score, or unavailable paired SOFA data. The archived screening log did not allow reliable reconstruction of mutually exclusive counts for each criterion. Figure 1 therefore reports the verified aggregate of 177 exclusions and lists the recorded categories without assigning unverified category-specific counts. The final analytic cohort included 1,023 patients: 513 with type 2 diabetes mellitus and 510 without type 2 diabetes mellitus. We did not perform a formal *a priori* sample-size calculation because all eligible patients during the study period were included. The primary T2DM model included four prespecified predictors and 154 in-hospital deaths, yielding 38.5 events per predictor parameter. The non-T2DM model used the same four-predictor structure and included 130 deaths, yielding 32.5 events per predictor parameter. These event ratios supported the prespecified model structure, although events per variable metrics represent only one consideration in sample-size planning for clinical prediction models (10). Figure 1 summarizes patient selection.

**Figure 1:**
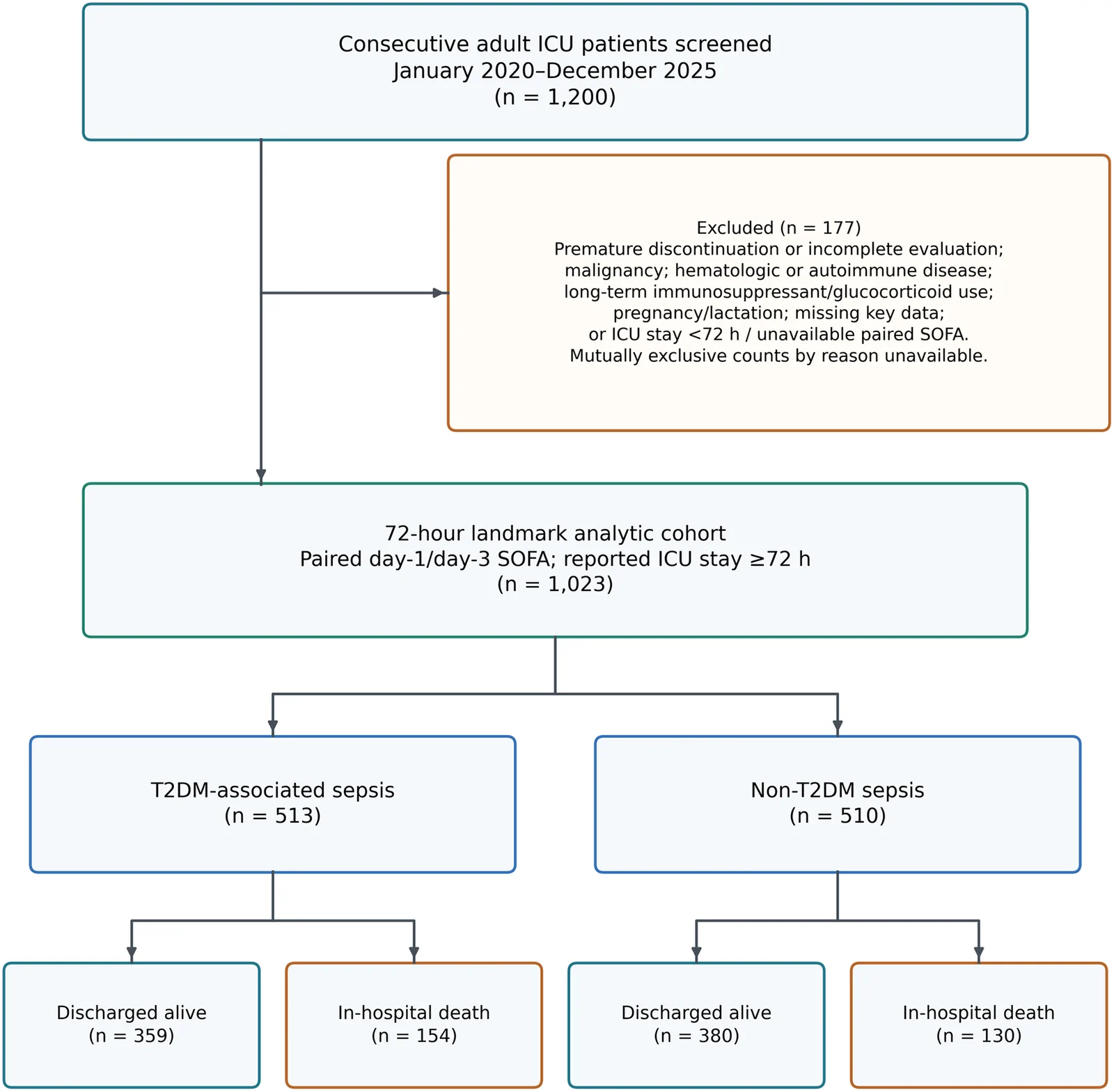
Study flow diagram. We screened consecutive patients from January 2020 through December 2025. The 72-h landmark cohort comprised 1,023 patients who remained in the ICU for at least 72 h and had paired day-1/day-3 SOFA measurements. The verified aggregate number of exclusions was 177. The diagram lists the recorded exclusion categories; mutually exclusive counts for individual categories could not be reconstructed reliably from the archived screening log and are therefore not reported.

### Data sources and record review

All source records were electronic. Medical Records Department staff retrospectively retrieved data from the hospital information system (HIS) using a prespecified extraction protocol that defined the variables, allowable assessment windows, and extraction criteria. Three ICU physicians—Dongxu Pan and Bo Zhang, both associate chief physicians, and Zongnan Lv, an attending physician—then reviewed the clinical and laboratory dataset for completeness and consistency with those definitions and time windows. Any questionable or internally inconsistent value was checked against the source electronic medical record. The revision-level audit confirmed the internal consistency of the core model variables. The original APACHE II scores were verified as integers, and the associated statistical results were checked. However, the archived audit trail did not record the investigator responsible for each field or an independent duplicate-abstraction process. We therefore could not calculate formal interrater agreement, and residual extraction or transcription errors cannot be excluded.

The reported dataset did not include variables identifying causative pathogens or community vs. hospital-acquired infections, and it did not provide a sufficiently granular classification of chronic comorbidities beyond T2DM and the prespecified exclusion criteria. These variables were therefore not incorporated into the comparative tables or prediction models. The eligibility criteria excluded malignant neoplasms, hematological disorders, autoimmune diseases, long-term immunosuppressive therapy, and systemic glucocorticoid use.

### Clinical data and immunological assessment

Demographic data, presence of an artificial airway, HbA1c, random blood glucose, cytokine concentrations, serum creatinine, blood urea nitrogen, lactate, procalcitonin (PCT), the neutrophil-to-lymphocyte ratio (NLR), SOFA score, and APACHE II score were collected within 24 h after ICU admission. SOFA scores were recorded on ICU days 1 and 3, and ΔSOFA was calculated as the day-3 minus day-1 SOFA using only paired observed values. The primary analysis used complete cases in the final 72-h landmark cohort. None of the analyzed variables was missing among the 1,023 included patients; therefore, no imputation was required in this cohort. Variable-level partial data among excluded patients could not be reconstructed reliably. As a result, we could not assess the missing-completely-at-random assumption for those exclusions or perform multiple imputation of the excluded records.

All SOFA values were checked against the 0–24-point theoretical scale, with organ subscore consistency reviewed as indicated.

For all 1,023 included patients, peripheral blood Th1 and Th2 cell proportions were quantified by flow cytometry using samples obtained within 24 h after ICU admission. For Th1/Th2 phenotyping, peripheral blood mononuclear cells were stained with fluorochrome-conjugated monoclonal antibodies against CD3, CD4, intracellular interferon-γ (IFN-γ), and intracellular interleukin-4 (IL-4), in accordance with the manufacturers' instructions and the hospital laboratory's standard operating procedures. Lymphocytes were first identified according to forward- and side-scatter characteristics, followed by sequential gating on singlet cells and CD3+CD4+ T lymphocytes. Th1 and Th2 cells were defined as CD3+CD4+IFN-γ+ and CD3+CD4+IL-4+ cells, respectively. The Th1/Th2 ratio was calculated as the percentage of Th1 cells divided by the percentage of Th2 cells among CD4+ T lymphocytes, with appropriate compensation settings and negative controls applied during acquisition and analysis. The analysis export retained the percentage-derived Th1/Th2 ratio, but not the separate Th1 and Th2 percentages or absolute cell counts. Absolute CD3+CD4+, Th1, and Th2 counts could not be reconstructed or linked reliably to the flow-cytometry gates.

Cytokines were measured in the clinical laboratory using standardized institutional procedures, and laboratory staff were blinded to clinical outcomes during assay processing. Routine internal quality control was performed for immunological assays according to accredited laboratory standards, but patient-level run-specific coefficients of variation were not captured in the retrospective dataset.

During the study period, the ICU implemented a standardized glycemic management protocol. In patients with hyperglycemia requiring insulin, regular insulin was administered by continuous intravenous infusion and titrated according to serial blood glucose measurements, targeting a blood glucose range of 8–10 mmol/L (144–180 mg/dL), consistent with international sepsis guidance (3). Patient-level insulin doses, serial glucose profiles, hypoglycemic episodes, and protocol adherence were not recorded in the extracted analytic dataset and could not be included as adjustment covariates.

### Outcome and model predictors

The primary outcome was all-cause death during the index hospital admission; in-hospital mortality was coded as 1; discharge alive from the hospital was coded as 0. Clinical improvement or transfer out of the ICU alone did not define survival. The term “discharged alive” is used consistently in Tables 1, 2 and Figures 1,2 for this final hospital disposition category. The outcome was abstracted from the final hospital disposition; an independent blinded outcome adjudication procedure was not documented in the available audit record. The length-of-stay variable represented ICU days, not time to final hospital death or live discharge. The prespecified Model 3 assessed whether Th1/Th2, IL-6, and ΔSOFA added prognostic information beyond day-1 SOFA. To avoid redundancy among overlapping severity measures, APACHE II, lactate, NLR, and day-3 SOFA were examined descriptively and in univariable or sensitivity analyses rather than entered together. *Post hoc* benchmarks included age alone, age plus day-1 SOFA, and Model 3 plus age. We also fitted a pooled secondary model that included diabetes status and age and used a global likelihood ratio test to evaluate diabetes-by-predictor interactions. Diabetes status was included because the purpose of this analysis was to determine whether cohort membership modified the associations of the four Model 3 predictors. We considered but did not add random glucose or HbA1c. Admission glucose is strongly affected by acute stress and treatment, whereas HbA1c contributed to the clinical classification of T2DM; including either variable would address a different metabolic prediction question and could introduce collinearity. This choice should not be interpreted as evidence that either measure lacks prognostic value. For coefficient reporting, age was scaled per 10-year increase and IL-6 per 100-pg/mL increase.

**Table 1:** Clinical and immunological characteristics by outcome in patients with T2DM and sepsis.

Variable	Discharged alive (*n* = 359)	In-hospital death (*n* = 154)	*P*-value
Age, years, median [IQR]	70 [64, 76]	74 [68, 80]	<0.001
APACHE II, median [IQR]	19 [16, 22]	30 [20, 38]	<0.001
day-1 SOFA, median [IQR]	9.00 [7.00, 12.00]	15.00 [12.00, 18.00]	<0.001
day-3 SOFA, median [IQR]	8.00 [6.00, 13.00]	16.00 [13.00, 19.00]	<0.001
ΔSOFA, median [IQR]	-1.00 [−2.00, 1.00]	2.00 [0.00, 5.00]	<0.001
Th1/Th2, median [IQR]	0.75 [0.58, 0.95]	1.50 [0.89, 2.53]	<0.001
IL-6, pg/mL, median [IQR]	325.81 [132.46, 521.98]	1094.16 [438.00, 2212.81]	<0.001
IL-10, pg/mL, median [IQR]	86.09 [44.23, 131.21]	266.24 [110.74, 513.53]	<0.001
NLR, median [IQR]	7.31 [5.48, 9.30]	14.49 [8.27, 24.64]	<0.001
Lactate, mmol/L, median [IQR]	4.63 [3.19, 6.42]	11.13 [5.53, 18.23]	<0.001
Serum creatinine, μmol/L, median [IQR]	156.50 [104.65, 209.35]	172.60 [117.90, 233.83]	0.035
Random blood glucose, mmol/L, median [IQR]	15.60 [13.65, 17.95]	16.35 [14.35, 18.58]	0.016
HbA1c, %, median [IQR]	8.50 [7.70, 9.55]	8.80 [7.50, 9.60]	0.589
ICU length of stay, days, median [IQR]	12 [7.5, 17]	13 [9, 17.75]	0.066
Artificial airway, *n* (%)	159 (44.3)	109 (70.8)	<0.001

Day-1 SOFA and day-3 SOFA were within the 0–24-point theoretical range. ΔSOFA = day-3 SOFA−day-1 SOFA and was calculated from paired observed values without imputation.

**Table 2:** Clinical and immunological characteristics by outcome in patients with sepsis but without T2DM.

Variable	Discharged alive (*n* = 380)	In-hospital death (*n* = 130)	*P*-value
Age, years, median [IQR]	66 [61, 72]	71 [65, 76]	0.002
APACHE II, median [IQR]	16 [14, 19]	30 [18, 35]	<0.001
day-1 SOFA, median [IQR]	7.00 [5.00, 10.00]	13.00 [10.00, 16.00]	<0.001
day-3 SOFA, median [IQR]	7.00 [4.00, 12.00]	14.00 [11.00, 18.00]	<0.001
ΔSOFA, median [IQR]	-1.00 [−2.00, 0.00]	2.00 [1.00, 5.00]	<0.001
Th1/Th2, median [IQR]	0.76 [0.59, 0.94]	1.67 [0.88, 2.50]	<0.001
IL-6, pg/mL, median [IQR]	162.76 [73.88, 274.15]	677.18 [231.52, 1107.46]	<0.001
IL-10, pg/mL, median [IQR]	60.99 [36.75, 86.55]	184.13 [75.61, 295.52]	<0.001
NLR, median [IQR]	6.72 [5.13, 8.37]	14.97 [7.74, 22.18]	<0.001
Lactate, mmol/L, median [IQR]	4.19 [3.04, 5.45]	10.22 [4.84, 15.18]	<0.001
Serum creatinine, μmol/L, median [IQR]	123.00 [83.93, 165.25]	135.45 [87.40, 180.88]	0.066
Blood urea nitrogen, mmol/L, median [IQR]	16.05 [10.08, 23.43]	18.15 [12.53, 26.88]	0.024
Artificial airway, *n* (%)	177 (46.6)	91 (70.0)	<0.001

SOFA-related variables were checked within the 0–24-point theoretical range; values in non-survivors did not show implausible clustering near the upper limit of the scale.

**Figure 2:**
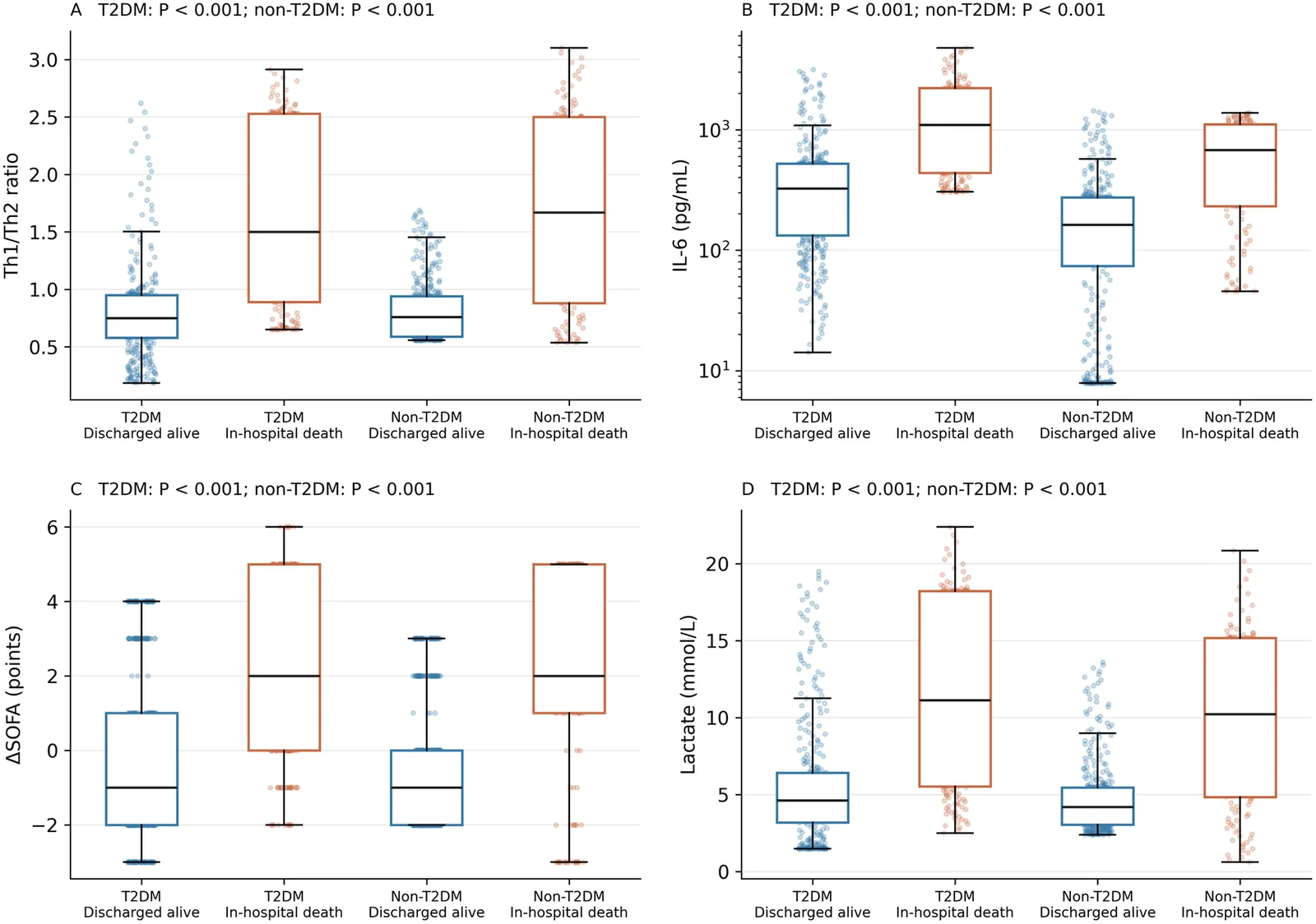
Outcome-stratified distributions of (A) the Th1/Th2 ratio, (B) IL-6, (C) ΔSOFA, and (D) lactate in the T2DM and non-T2DM sepsis cohorts, regenerated from the supplied analytic cohorts. “Discharged alive” denotes final discharge alive from the index hospital admission; ICU transfer or clinical improvement alone does not qualify. “In-hospital death” denotes death during that admission. Boxes show the median and interquartile range, whiskers extend to 1.5 times the interquartile range, and points represent individual observations without identifiers. IL-6 is displayed on a logarithmic axis. *P* values compare final outcome groups within each cohort using two-sided Mann–Whitney *U* tests.

### Statistical analysis

Normally distributed continuous variables were summarized as mean ± standard deviation and compared with the two-sided Student independent-samples *t* test. Skewed continuous variables were summarized as median [interquartile range] and compared with the two-sided Mann–Whitney *U*-test with tie and continuity corrections. Quartiles were calculated by linear interpolation (type 7), and rounding was applied only after statistical computation. Categorical variables were reported as counts and percentages and compared with the Yates-corrected *χ*^2^ test. For in-hospital mortality, univariable logistic regression and receiver operating characteristic (ROC) analyses were used to assess the discriminatory performance of candidate continuous predictors, with areas under the ROC curve (AUCs) and 95% confidence intervals reported.

Univariable and multivariable logistic regression models were fitted with in-hospital mortality as the dependent variable. In Tables 3, 4, continuous predictors were standardized using the T2DM cohort mean and sample standard deviation (SD; denominator n−1). Unpenalized maximum-likelihood logistic regression was used, with odds ratios reported per 1−SD increase (OR/SD). Model-based standard errors were obtained from the inverse observed information matrix; 95% confidence intervals were calculated as exp[*β* ± 1.959964  ×  SE(*β*)], and two-sided *P*-values were obtained using Wald z-tests. These coefficient intervals are distinct from the bootstrap optimism correction of predictive performance. Sample SDs were also used in descriptive summaries. Complete original-scale coefficients and intercepts are reported in Table 5. Model 1 = day-1 SOFA; Model 2 = day-1 SOFA + Th1/Th2 + IL-6; Model 3=day-1 SOFA + ΔSOFA + Th1/Th2 + IL-6. Secondary benchmarks were age alone, age+Model 1, Model 3+age, and pooled Model 3+diabetes status+age. Models 1 and 2 use baseline measurements, but their reported performance remains conditional on membership in the 72-h landmark cohort; Model 3 is a 72-h landmark reassessment model. APACHE II was recorded as an integer score and was used in descriptive, univariable logistic regression and ROC analyses. APACHE II was not included in Models 1-3 or 3a.

**Table 3:** Univariable logistic regression in the T2DM-associated sepsis cohort.

Variable	OR/SD	95% CI	*P*-value
Age	1.54	1.27–1.87	<0.001
day-1 SOFA	3.21	2.55–4.04	<0.001
day-3 SOFA	3.40	2.68–4.30	<0.001
ΔSOFA	2.31	1.88–2.85	<0.001
Th1/Th2	4.06	3.09–5.34	<0.001
IL-6	3.21	2.46–4.17	<0.001
IL-10	3.04	2.41–3.85	<0.001
NLR	2.63	2.13–3.25	<0.001
Lactate	2.71	2.19–3.36	<0.001
APACHE II	2.92	2.34–3.63	<0.001

OR/SD is the odds ratio per 1-SD increase, using the T2DM cohort sample SD (denominator *n*−1). Each row is a separate unpenalized logistic model with an intercept, fitted to all 513 patients (154 deaths). Confidence intervals and *P*-values use model-based Wald inference. APACHE II was analyzed using the verified integer scores.

**Table 4:** Multivariable logistic regression predictors of in-hospital mortality in the T2DM-associated sepsis cohort.

Variable	Multivariable OR/SD	95% CI	*P*-value	Independent predictor
Day-1 SOFA	1.92	1.44–2.56	<0.001	Yes
Th1/Th2	2.78	1.95–3.95	<0.001	Yes
IL-6	1.56	1.10–2.21	0.012	Yes
ΔSOFA	1.71	1.29–2.27	<0.001	Yes

The multivariable model included day-1 SOFA, Th1/Th2, IL-6, and ΔSOFA. OR/SD was used to compare relative effect size across variables with different units. The nomogram and individualized predicted probabilities were derived from original-scale coefficients, not from standardized ORs. Standardization uses the cohort sample SD (denominator *n*−1), with model-based Wald confidence intervals and *P*-values, as in Table 3.

**Table 5:** Complete original-scale logistic regression coefficients and intercepts.

Cohort and model	Term	Coefficient *β*	SE	OR (95% CI)	*P*-value
T2DM-associated sepsis; Model 3	Intercept	-4.989546	0.503927	0.007 (0.003–0.018)	<0.001
T2DM-associated sepsis; Model 3	day-1 SOFA (per point)	0.160816	0.036368	1.174 (1.094–1.261)	<0.001
T2DM-associated sepsis; Model 3	ΔSOFA (per point)	0.207665	0.055150	1.231 (1.105–1.371)	<0.001
T2DM-associated sepsis; Model 3	Th1/Th2 (per unit)	1.562379	0.274756	4.770 (2.784–8.174)	<0.001
T2DM-associated sepsis; Model 3	IL-6 (per 100 pg/mL)	0.053955	0.021484	1.055 (1.012–1.101)	0.012
T2DM-associated sepsis; Model 3+age	Intercept	-7.828587	1.169645	0.000 (0.000–0.004)	<0.001
T2DM-associated sepsis; Model 3+age	day-1 SOFA (per point)	0.163085	0.036761	1.177 (1.095–1.265)	<0.001
T2DM-associated sepsis; Model 3+age	ΔSOFA (per point)	0.202703	0.055918	1.225 (1.098–1.367)	<0.001
T2DM-associated sepsis; Model 3+age	Th1/Th2 (per unit)	1.539735	0.276329	4.663 (2.713–8.015)	<0.001
T2DM-associated sepsis; Model 3+age	IL-6 (per 100 pg/mL)	0.052901	0.021879	1.054 (1.010–1.101)	0.016
T2DM-associated sepsis; Model 3+age	Age (per 10 years)	0.395949	0.141569	1.486 (1.126–1.961)	0.005
Non-T2DM sepsis; Model 3	Intercept	-4.238102	0.423273	0.014 (0.006–0.033)	<0.001
Non-T2DM sepsis; Model 3	day-1 SOFA (per point)	0.048357	0.038900	1.050 (0.972–1.133)	0.214
Non-T2DM sepsis; Model 3	ΔSOFA (per point)	0.221009	0.063112	1.247 (1.102–1.412)	<0.001
Non-T2DM sepsis; Model 3	Th1/Th2 (per unit)	2.149044	0.411360	8.577 (3.830–19.208)	<0.001
Non-T2DM sepsis; Model 3	IL-6 (per 100 pg/mL)	0.068562	0.046390	1.071 (0.978–1.173)	0.139
Non-T2DM sepsis; Model 3+age	Intercept	-6.346636	1.086635	0.002 (0.000–0.015)	<0.001
Non-T2DM sepsis; Model 3+age	day-1 SOFA (per point)	0.044027	0.039312	1.045 (0.968–1.129)	0.263
Non-T2DM sepsis; Model 3+age	ΔSOFA (per point)	0.218402	0.063483	1.244 (1.099–1.409)	<0.001
Non-T2DM sepsis; Model 3+age	Th1/Th2 (per unit)	2.174871	0.410905	8.801 (3.933–19.693)	<0.001
Non-T2DM sepsis; Model 3+age	IL-6 (per 100 pg/mL)	0.067756	0.046297	1.070 (0.977–1.172)	0.143
Non-T2DM sepsis; Model 3+age	Age (per 10 years)	0.313774	0.144856	1.369 (1.030–1.818)	0.030
Pooled landmark cohort; Pooled Model 3+DM+age	Intercept	-6.726541	0.769057	0.001 (0.000–0.005)	<0.001
Pooled landmark cohort; Pooled Model 3+DM+age	day-1 SOFA (per point)	0.115426	0.026122	1.122 (1.066–1.181)	<0.001
Pooled landmark cohort; Pooled Model 3+DM+age	ΔSOFA (per point)	0.225302	0.041318	1.253 (1.155–1.358)	<0.001
Pooled landmark cohort; Pooled Model 3+DM+age	Th1/Th2 (per unit)	1.708065	0.221335	5.518 (3.576–8.515)	<0.001
Pooled landmark cohort; Pooled Model 3+DM+age	IL-6 (per 100 pg/mL)	0.053689	0.019714	1.055 (1.015–1.097)	0.006
Pooled landmark cohort; Pooled Model 3+DM+age	T2DM status (1 vs. 0)	-0.338393	0.203567	0.713 (0.478–1.062)	0.096
Pooled landmark cohort; Pooled Model 3+DM+age	Age (per 10 years)	0.343123	0.099956	1.409 (1.159–1.714)	<0.001

Coefficients use SOFA and ΔSOFA per point, Th1/Th2 per unit, IL-6 per 100 pg/mL, age per 10 years, and T2DM status coded 1 for T2DM and 0 otherwise. Predicted probability=1/[1+exp(−linear predictor)].

Discrimination was assessed using ROC curves and DeLong 95% confidence intervals and comparisons; therefore, the day-1 SOFA AUC and confidence interval are identical wherever Model 1 is reported in Tables 6, 7. Calibration was evaluated with the Brier score, calibration intercept and slope estimated by regressing the outcome on the logit of predicted risk, grouped calibration plots, and the Hosmer–Lemeshow goodness-of-fit test. Internal validation was performed using 1,000 non-parametric optimism-bootstrap resamples. If D_app denotes apparent performance, D_b,boot performance in bootstrap sample b, and D_b,orig performance when that bootstrap-fitted model was applied to the original cohort, mean optimism was O=mean(D_b,boot−D_b,orig), and the corrected point estimate was D_corr=D_app−O. The same framework was applied to the Brier score, calibration intercept, and calibration slope. Because apparent logistic-model calibration intercepts and slopes are 0 and 1 by construction in the development data, Table 7 emphasizes the optimism-corrected values. For internal validation, all coefficients in each fixed model specification were refitted in 1,000 patient-level bootstrap samples. Seeds were 20260827 for T2DM, 20260828 for non-T2DM, and 20260829 for the pooled cohort; the same draws were used across models within a cohort. Calibration intercept and slope were estimated jointly from the original-cohort outcomes and each bootstrap model's predicted log odds. No imputation, predictor selection, or hyperparameter tuning was performed within these fixed-specification fits. All resamples converged. DeLong intervals are reported for apparent AUCs; confidence intervals were not estimated for the optimism-corrected point estimates. Figure 3 compares apparent and optimism-corrected AUC point estimates. Given the event counts and the objective of quantifying model optimism and calibration shrinkage rather than reporting discrimination from a single held-out partition, bootstrap resampling was selected for internal validation. We considered but did not retain a single train–test split because it would reduce the information available for development and make the performance estimate dependent on one random partition. This choice does not imply that bootstrap validation estimates final external performance: it remains internal validation and cannot substitute for a temporally separated or geographically external cohort. Clinical utility was explored using empirical decision curve analysis across threshold probabilities from 0.05 to 0.80 in 0.01 increments, with net benefit compared against Models 1 and 2 and treat-all and treat-none strategies (11, 12). Variance inflation factors were examined for multicollinearity. No resampling or weighting was used to address class imbalance, and no fairness adjustment was applied. Model performance was not assessed across sociodemographic groups because the necessary variables were unavailable.

**Table 6:** Univariable discriminatory performance of key indicators for in-hospital mortality.

Variable	T2DM AUC (95% CI)	*P*-value	Non-T2DM sepsis AUC (95% CI)	*P*-value
Th1/Th2	0.812 (0.771–0.853)	<0.001	0.792 (0.737–0.848)	<0.001
IL-6	0.805 (0.767–0.844)	<0.001	0.768 (0.717–0.820)	<0.001
IL-10	0.798 (0.759–0.837)	<0.001	0.754 (0.700–0.807)	<0.001
Lactate	0.784 (0.741–0.826)	<0.001	0.773 (0.714–0.833)	<0.001
NLR	0.779 (0.736–0.822)	<0.001	0.769 (0.720–0.818)	<0.001
day-1 SOFA	0.781 (0.740–0.822)	<0.001	0.761 (0.719–0.804)	<0.001
day-3 SOFA	0.811 (0.774–0.848)	<0.001	0.785 (0.740–0.830)	<0.001
ΔSOFA	0.735 (0.691–0.779)	<0.001	0.718 (0.655–0.781)	<0.001
APACHE II	0.789 (0.749–0.829)	<0.001	0.781 (0.729–0.834)	<0.001

In-hospital mortality was the positive outcome. All apparent AUC 95% confidence intervals were calculated with the DeLong method. Consequently, the day-1 SOFA Model 1 estimates are identical in Tables 6, 7. AUC > 0.5 indicates discrimination better than chance. APACHE II results were calculated using the verified integer scores.

**Table 7:** Apparent and internally validated performance of the mortality prediction models.

Cohort	Model	Apparent AUC (DeLong 95% CI)	Corrected AUC	App./corr. Brier	Corrected intercept	Corrected slope	Apparent HL P
T2DM-associated sepsis	Model 1	0.781 (0.740–0.822)	0.782	0.161/0.162	0.001	1.003	<0.001
T2DM-associated sepsis	Model 2	0.838 (0.798–0.878)	0.837	0.125/0.127	−0.027	0.979	0.005
T2DM-associated sepsis	Model 3	0.861 (0.827–0.895)	0.858	0.123/0.125	−0.034	0.971	0.013
T2DM-associated sepsis	Model 3+age	0.870 (0.837–0.902)	0.865	0.121/0.124	−0.042	0.962	0.014
Non-T2DM sepsis	Model 1	0.761 (0.719–0.804)	0.760	0.156/0.157	0.005	0.999	<0.001
Non-T2DM sepsis	Model 2	0.821 (0.774–0.868)	0.816	0.117/0.119	−0.022	0.978	0.043
Non-T2DM sepsis	Model 3	0.848 (0.806–0.890)	0.841	0.114/0.118	−0.035	0.962	0.227
Non-T2DM sepsis	Model 3+age	0.848 (0.806–0.891)	0.838	0.112/0.116	−0.045	0.951	0.267

Model 1=day-1 SOFA; Model 2 adds Th1/Th2 and IL-6; Model 3 adds ΔSOFA; Model 3+age is a *post hoc* secondary benchmark. Apparent AUC confidence intervals use DeLong's method. Corrected AUC, Brier, calibration intercept, and calibration slope were recomputed as apparent performance minus mean training-vs.-original-cohort optimism from 1,000 bootstrap refits of each fixed model. All refits converged. The intercept and slope are jointly estimated calibration parameters. Apparent intercept and slope equal 0 and 1 by construction. Corrected point estimates have no confidence intervals; the previous nested-bootstrap intervals are withdrawn. Monte Carlo variation can produce small negative estimated optimism. HL uses up to 10 distinct risk groups. The significant T2DM Model 3 HL result (*P*=0.013) cautions against claiming adequate local calibration.

**Figure 3:**
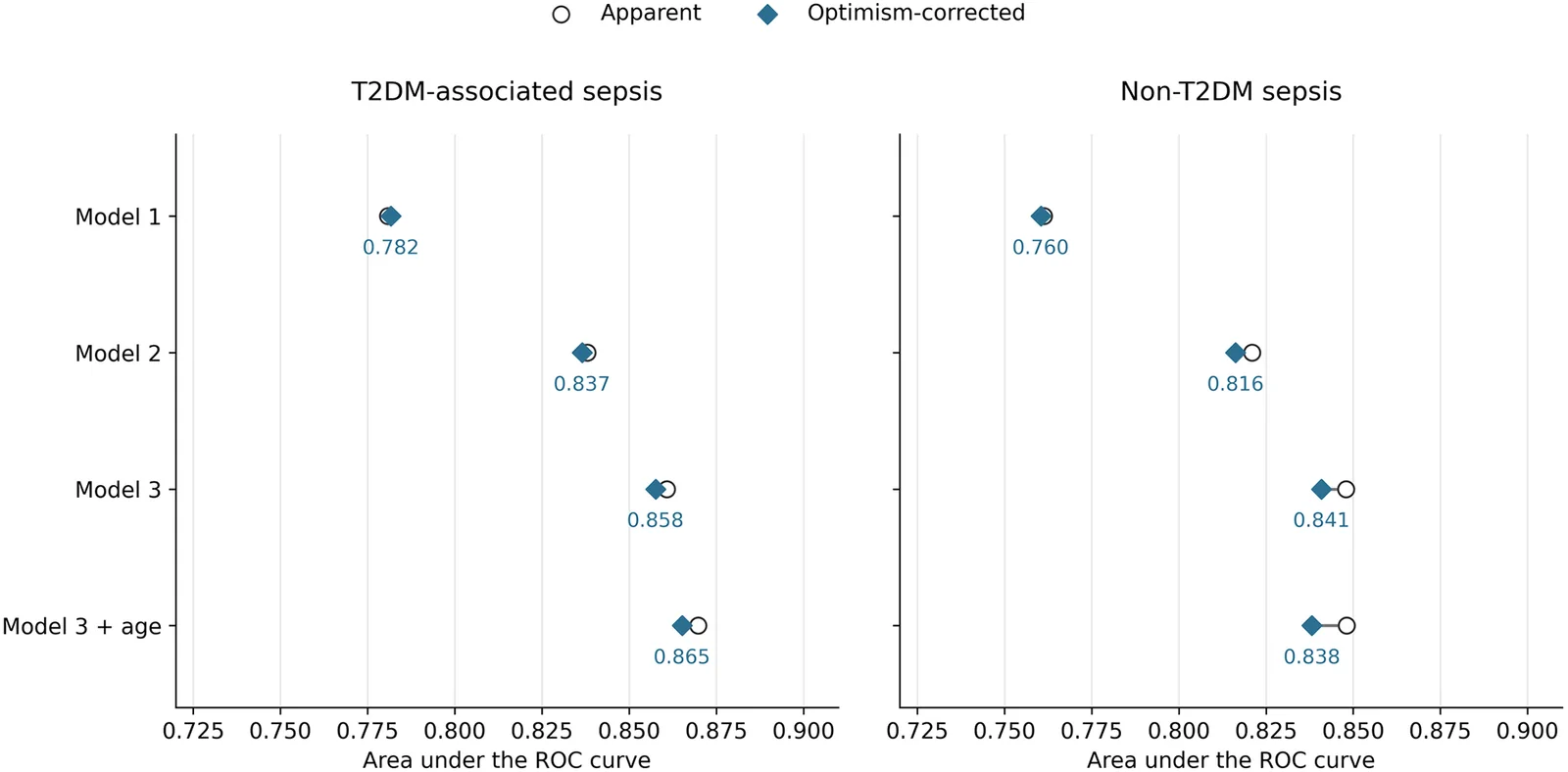
Apparent and optimism-corrected AUC point estimates from the recomputed internal validation. Each fixed model was refitted in 1,000 patient-level bootstrap resamples. Open circles show apparent AUC and filled diamonds show apparent AUC minus mean optimism; connecting lines indicate the correction, not a confidence interval. No sampling distributions or confidence intervals for corrected AUC are displayed. Model 3+age is a *post hoc* benchmark. Full replicate-level aggregate performance is provided in Supplementary File 1.

Because ΔSOFA is derived from the day-3 minus day-1 SOFA, Model 3a (day-3 SOFA+Th1/Th2+IL-6) was compared with Model 3 using AUC, Brier score, Hosmer–Lemeshow goodness-of-fit testing, and paired DeLong testing. DeLong comparisons describe AUC differences in the development sample and are not used alone to establish incremental value for models fitted and evaluated in the same patients. Model 3a is the constrained version of Model 3 in which the day-1 SOFA and ΔSOFA coefficients are equal; a one-degree-of-freedom likelihood ratio test was therefore added for this sensitivity comparison. Valid times to hospital death and final discharge alive were unavailable; ICU length of stay was not substituted for these times. Cox and Fine–Gray competing-risks analyses are consequently not reported. The original analyses used IBM SPSS Statistics version 27.0 (IBM Corp., Armonk, NY, USA) and R version 4.6.1 (R Foundation for Statistical Computing, Vienna, Austria). During peer-review revisions, an independent audit refitted Models 1–3 and 3a and checked their apparent ROC, Brier, Hosmer–Lemeshow, and paired-comparison results using Python 3.12.13, NumPy 2.3.5, and SciPy 1.17.0; the verification code is supplied as Supplementary File 1. The present correction additionally recomputed Tables 1–4, 6, 8 and the directly related descriptive results; each of the 10 univariable fits in Table 3 was cross-checked using an independent BFGS optimizer. The internal validation analysis used 1,000-resample optimism correction for Models 1–3 and 3a, Model 3 plus age in each cohort, and pooled Model 3 plus diabetes status and age. Bootstrap replicate-level aggregate performance and executable code are provided (Supplementary File 1). These internal estimates remain conditional on the fixed model specifications and the selected 72-h cohorts. All tests were two-sided with *P* < 0.05. Generative AI was not used to generate, alter, or impute study data; its role in editorial and analytical assistance is disclosed in the Acknowledgments.

**Table 8:** Baseline clinical and immunological characteristics of septic patients with and without T2DM.

Variable	T2DM-associated sepsis (*n* = 513)	Non-T2DM sepsis (*n* = 510)	*P*-value
Male sex, *n* (%)	322 (62.8)	306 (60.0)	0.398
In-hospital death, *n* (%)	154 (30.0)	130 (25.5)	0.122
Artificial airway, *n* (%)	268 (52.2)	268 (52.5)	0.971
Age, years, mean ± SD	71 ± 9	67 ± 9	<0.001
day-1 SOFA, median [IQR]	10.00 [7.00, 15.00]	9.00 [5.00, 13.00]	<0.001
day-3 SOFA, median [IQR]	10.00 [7.00, 16.00]	9.00 [5.00, 13.00]	<0.001
ΔSOFA, median [IQR]	0.00 [−2.00, 3.00]	0.00 [−2.00, 2.00]	0.421
APACHE II, median [IQR]	20 [17, 25]	17 [14, 22]	<0.001
ICU length of stay, days, median [IQR]	12 [8, 17]	10 [7, 15]	<0.001
NLR, mean ± SD	10.25 ± 6.57	9.17 ± 5.73	0.005
Lactate, mmol/L, mean ± SD	7.11 ± 5.25	5.93 ± 4.04	<0.001
PCT, ng/mL, median [IQR]	8.68 [3.86, 14.31]	6.02 [3.09, 10.32]	<0.001
Serum creatinine, μmol/L, median [IQR]	161.10 [109.30, 221.30]	123.95 [84.70, 170.85]	<0.001
Blood urea nitrogen, mmol/L, mean ± SD	22.77 ± 12.90	17.70 ± 9.81	<0.001
Random blood glucose, mmol/L, median [IQR]	16.00 [13.80, 18.10]	11.00 [8.90, 13.20]	<0.001
HbA1c, %, median [IQR]	8.60 [7.70, 9.60]	5.80 [5.40, 6.40]	<0.001
Th1/Th2, median [IQR]	0.83 [0.63, 1.14]	0.83 [0.64, 1.14]	0.454
IL-6, pg/mL, median [IQR]	399.37 [216.33, 731.37]	202.44 [101.05, 363.88]	<0.001
IL-10, pg/mL, median [IQR]	105.69 [59.24, 176.79]	69.74 [42.20, 111.07]	<0.001
IL-12p70, pg/mL, median [IQR]	31.59 [19.25, 44.24]	21.78 [14.21, 31.44]	<0.001
IFN-*γ*, pg/mL, median [IQR]	48.81 [27.30, 69.10]	33.66 [20.91, 49.73]	<0.001
TNF-α, pg/mL, median [IQR]	73.21 [47.91, 110.60]	50.49 [28.35, 71.58]	<0.001
IL-4, pg/mL, median [IQR]	26.72 [17.72, 37.71]	18.43 [12.22, 26.01]	<0.001
IL-1β, pg/mL, median [IQR]	63.17 [37.83, 87.81]	43.57 [26.58, 61.05]	<0.001

Skewed variables are shown as median [IQR], normally distributed variables as mean ± SD, and categorical variables as *n* (%). SOFA scores were quality-controlled within the theoretical 0–24-point range and are presented uniformly as median [IQR]. *P*-values use Student *t*-tests, Mann–Whitney *U*-tests, or Yates-corrected *χ*^2^ tests according to the summary type. APACHE II was analyzed using the verified integer scores.

## Results

### Baseline two-cohort characteristics: increased immunometabolic and inflammatory burden in patients with T2DM

Of the 1,200 patients screened, 177 were excluded according to the predefined eligibility criteria, leaving 1,023 patients with Sepsis-3-defined sepsis for the final analysis: 513 patients with T2DM and 510 patients without T2DM. In-hospital death occurred in 154 patients (30.0%) in the T2DM sepsis cohort and in 130 patients (25.5%) in the non-T2DM sepsis cohort. The crude mortality difference was not statistically significant (*P* = 0.122). This absolute difference of 4.5 percentage points should not be taken as evidence that diabetes has no effect on mortality, because the study was not specifically powered for between-cohort mortality comparisons. Patient selection and cohort allocation are shown in Figure 1.

Sex distribution and the proportion of patients with an artificial airway were similar between the cohorts. Compared with patients without T2DM, those in the T2DM sepsis cohort were older and had higher blood glucose, HbA1c, inflammatory cytokine levels, creatinine, blood urea nitrogen, NLR, lactate, PCT, ICU length of stay, and APACHE II scores. After SOFA quality control, day-1 and day-3 SOFA values in both cohorts remained within the 0–24-point theoretical range and were higher in the T2DM cohort. Overall, patients with T2DM exhibited a broader immunometabolic and inflammatory burden (Table 8).

### Clinical and immunological characteristics by outcome in the T2DM sepsis cohort

Within the T2DM sepsis cohort, non-survivors were older and demonstrated higher APACHE II scores, day-1 SOFA, day-3 SOFA, ΔSOFA, the Th1/Th2, IL-6, IL-10, NLR, lactate, serum creatinine, random blood glucose, and a higher proportion requiring an artificial airway than survivors (all *P* < 0.05). Among non-survivors, day-1 SOFA was 15.00 [12.00, 18.00] and day-3 SOFA was 16.00 [13.00, 19.00], indicating severe organ dysfunction while remaining consistent with the expected distribution and theoretical range. HbA1c and ICU length of stay did not differ significantly between outcome groups (Table 1).

### Clinical and immunological characteristics by outcome in the non-T2DM sepsis cohort

In the non-T2DM sepsis cohort, non-survivors likewise had higher values for the Th1/Th2, IL-6, IL-10, NLR, lactate, day-1 SOFA, day-3 SOFA, and ΔSOFA than survivors. These findings suggest that immune imbalance and worsening organ dysfunction during the first 72 h are common features of fatal sepsis. The overall cytokine and glycometabolic burden was lower than in the type 2 diabetes mellitus cohort, but the direction of the risk indicators was similar (Table 2). The outcome-stratified distributions of the Th1/Th2 ratio, IL-6, ΔSOFA, and lactate are shown in Figure 2.

### Univariable discrimination of key indicators for in-hospital mortality

Univariable logistic regression and ROC analyses showed that the Th1/Th2 ratio, IL-6, IL-10, lactate, the NLR, day-1 SOFA, day-3 SOFA, and ΔSOFA significantly discriminated in-hospital mortality in both cohorts. APACHE II yielded AUCs of 0.789 (95% CI, 0.749–0.829) in the T2DM cohort and 0.781 (95% CI, 0.729–0.834) in the non-T2DM cohort (Table 6). These APACHE II findings remain provisional pending source record verification of the fractional values.

### Univariable associations and prespecified multivariable modeling

The univariable results supported the prespecified staged modeling strategy. We retained day-1 SOFA as the baseline severity measure and sequentially added the Th1/Th2 ratio, IL-6, and ΔSOFA. APACHE II, day-3 SOFA, lactate, and the NLR were not entered together because they capture overlapping aspects of illness severity. *Post hoc* analyses then evaluated age alone, age plus SOFA, Model 3 plus age, and the age-adjusted pooled model that included diabetes status.

In the T2DM Model 3, day-1 SOFA (OR/SD, 1.92; 95% CI, 1.44–2.56), ΔSOFA (1.71; 1.29–2.27), Th1/Th2 (2.78; 1.95–3.95), and IL-6 (1.56; 1.10–2.21) were independently associated with mortality. All VIF values were below 3. Complete original-scale coefficients, intercepts, and executable equations are provided in Table 5.

### Performance of stepwise, age-benchmark, and pooled mortality models

In the T2DM cohort, apparent AUCs for Models 1, 2, and 3 were 0.781, 0.838, and 0.861. Paired development-sample DeLong comparisons demonstrated significant incremental discrimination for Model 2 vs. Model 1 (*P* < 0.001), Model 3 vs. Model 2 (*P* = 0.007), and Model 3 vs. Model 1 (*P* < 0.001). The same staged pattern occurred in the non-T2DM cohort (0.761, 0.821, and 0.848; corresponding *P*-values 0.003, 0.046, and <0.001). The corresponding ROC curves for Models 1-3 in both cohorts are shown in Figure 4.

**Figure 4:**
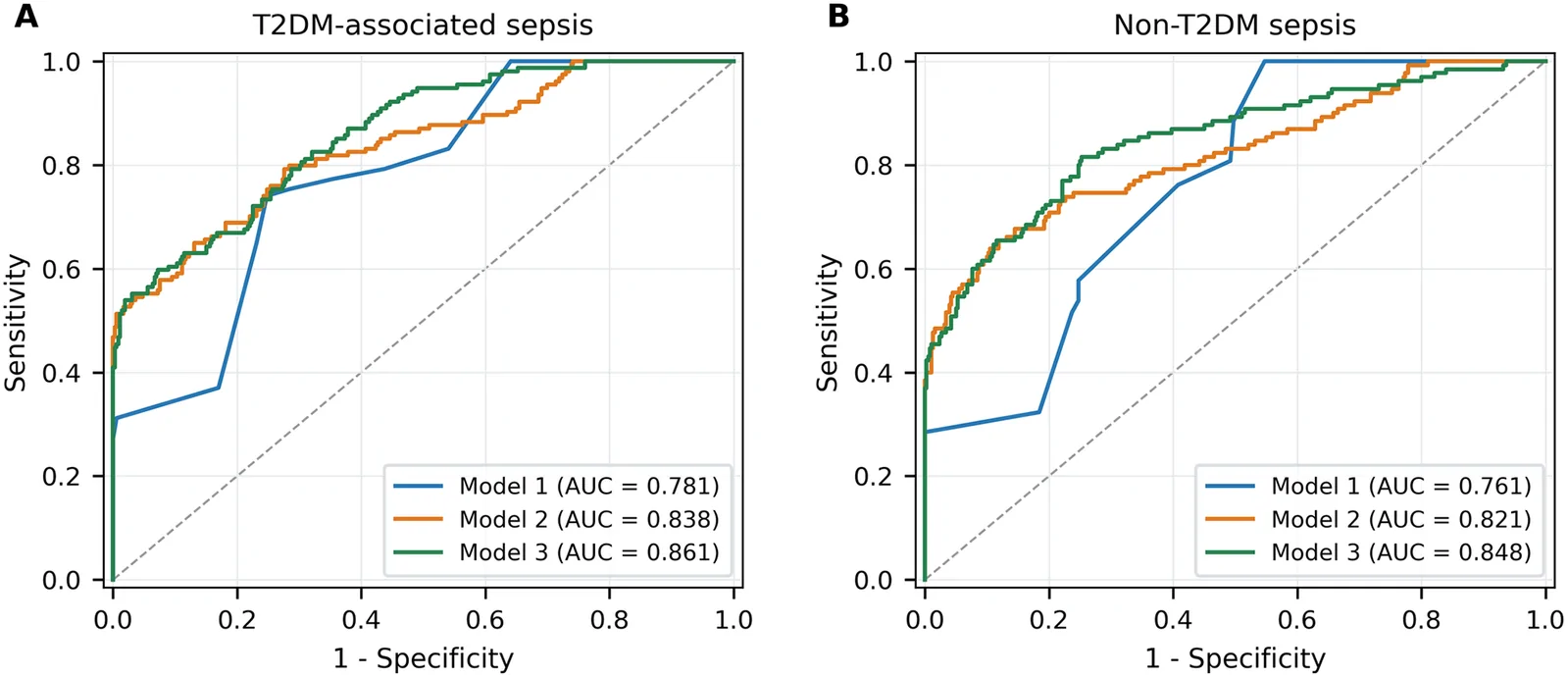
ROC curves of the three prespecified stepwise models for in-hospital mortality. **(A)** T2DM-associated sepsis; **(B)** non-T2DM sepsis. Curves were regenerated from the verified model probabilities and show apparent development-sample discrimination without patient-level numerical labels. All distinct thresholds and coordinates are provided in Supplementary Data Sheet 1. Recomputed optimism-corrected AUC point estimates are reported in Figure 3 and Table 7; no corrected-AUC confidence intervals are reported.

After 1,000 optimism-bootstrap resamples, the corrected Model 3 AUC was 0.858 in the T2DM cohort and 0.841 in the non-T2DM cohort. Apparent/corrected Brier scores were 0.123/0.125 and 0.114/0.118, respectively. The corrected calibration intercept/slope was −0.034/0.971 in T2DM and −0.035/0.962 in the non-T2DM cohort. Apparent Hosmer–Lemeshow *P* values were 0.013 and 0.227, respectively. The significant result in the T2DM cohort suggests possible groupwise or local miscalibration not fully captured by global intercepts and slopes. Figure 3 displays apparent and corrected AUC point estimates, and Table 7 reports the corresponding performance measures. Corrected point estimates do not represent external validation results, and confidence intervals for them are not reported.

Age alone was markedly less discriminative than Model 3, with an AUC of 0.634 (95% CI, 0.584–0.683) in the T2DM cohort and 0.591 (0.527–0.655) in the non-T2DM cohort. Adding age to Model 3 produced an apparent AUC of 0.870 and a corrected AUC of 0.865 in the T2DM cohort. The AUC increase over Model 3 was not statistically significant (DeLong *P* = 0.099), although the age coefficient was significant (*P* = 0.005) and the Brier score improved from 0.123 to 0.121. In the non-T2DM cohort, the apparent AUC was 0.848 for Model 3 plus age vs. 0.848 for Model 3 (*P* = 0.983), although the age coefficient was significant (*P* = 0.030). Thus, age remained independently associated with mortality but added little discrimination beyond the established multivariable model.

In the pooled cohort of 1,023 patients, Model 3 plus diabetes status and age yielded an apparent AUC of 0.862 and an optimism-corrected AUC of 0.857. The adjusted diabetes status coefficient was negative but did not reach conventional statistical significance (*P* = 0.096). The global likelihood ratio test for the four predictor-by-diabetes interactions was also non-significant (*P* = 0.191). These findings do not support the superiority of separate models. We therefore retained the cohort-specific models as parallel exploratory analyses and report the pooled model as a sensitivity benchmark.

For an individual patient, the predicted in-hospital mortality probability is *P* = 1/[1+exp(−LP)]. The T2DM Model 3 linear predictor is LP = −4.9895+0.1608(day-1 SOFA)+0.2077(ΔSOFA)+1.5624(Th1/Th2)+0.0005396(IL-6, pg/mL). The non-T2DM Model 3 linear predictor is LP=−4.2381+0.04836(day-1 SOFA)+0.2210(ΔSOFA)+2.1490(Th1/Th2)+0.0006856(IL-6, pg/mL). The age-adjusted pooled linear predictor is LP=−6.7265+0.1154(day-1 SOFA)+0.2253(ΔSOFA)+1.7081(Th1/Th2)+0.0005369(IL-6, pg/mL)−0.3384(T2DM status, 1=yes)+0.03431(age, years). The coefficient-based nomograms for the two cohort-specific Model 3 models are shown in Figure 5.

**Figure 5:**
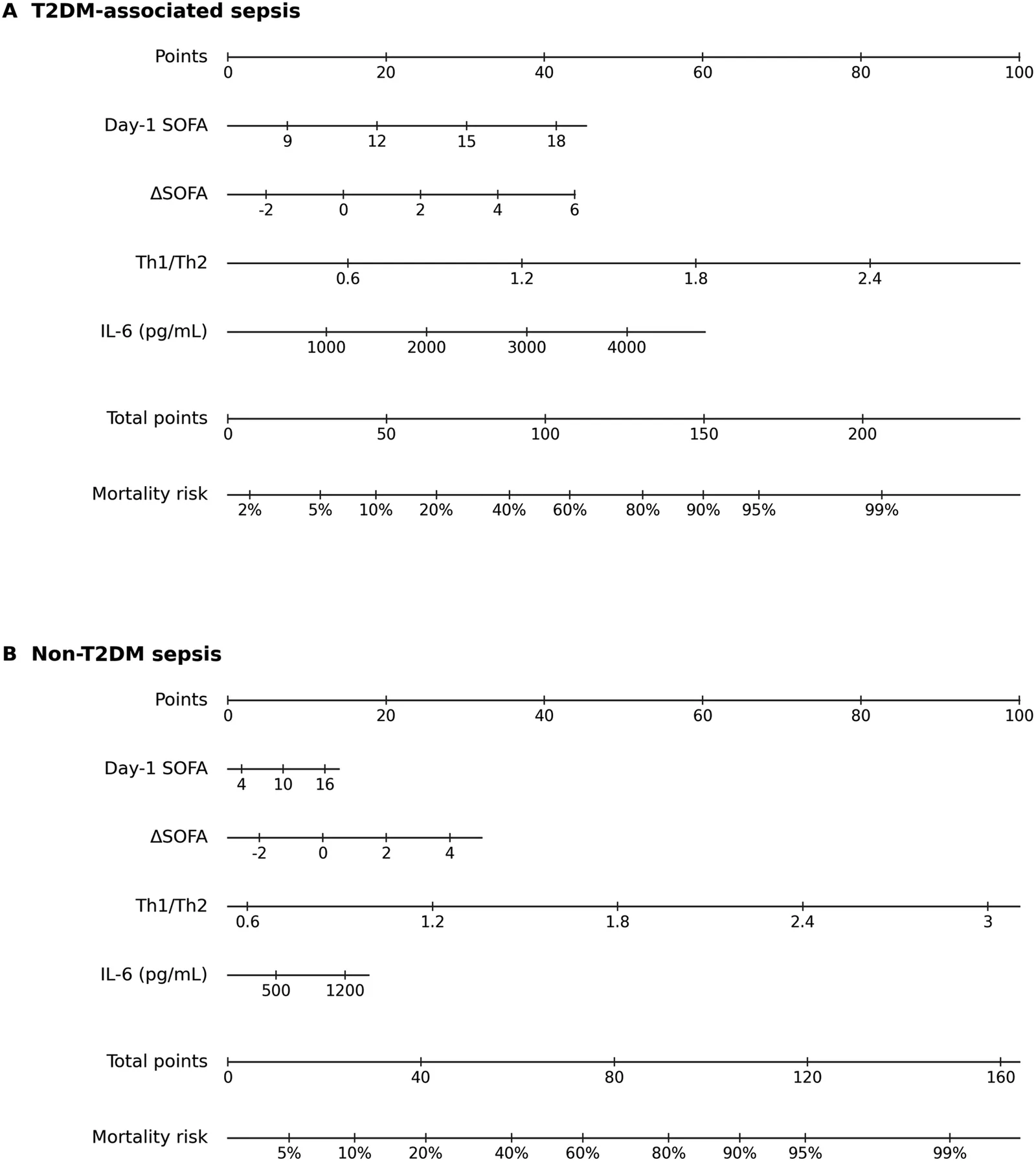
Coefficient-based nomograms for model 3. **(A)** T2DM-associated sepsis; **(B)** non-T2DM sepsis. Predictor axes use the observed ranges and original-scale logistic coefficients. Project each predictor value to the common points axis, sum the points, and align total points with mortality risk. The points-to-risk mapping reproduces the fitted logistic probabilities to numerical precision; individual patient labels are omitted. IL-6 is shown in pg/mL. Use the complete equations and intercepts in Table 5 for calculation. These graphical displays have not been externally validated for clinical use.

In the development dataset, Model 3 demonstrated the highest apparent net benefit among Models 1–3 and the treat-all and treat-none strategies across threshold probabilities of approximately 0.12–0.21 in the T2DM cohort and 0.13–0.23 in the non-T2DM cohort. In addition, less continuous regions of favorable net benefit were observed at higher thresholds (Figure 6). Because these ranges were derived from the development sample, they are exploratory and should not be interpreted as validated clinical decision thresholds.

**Figure 6:**
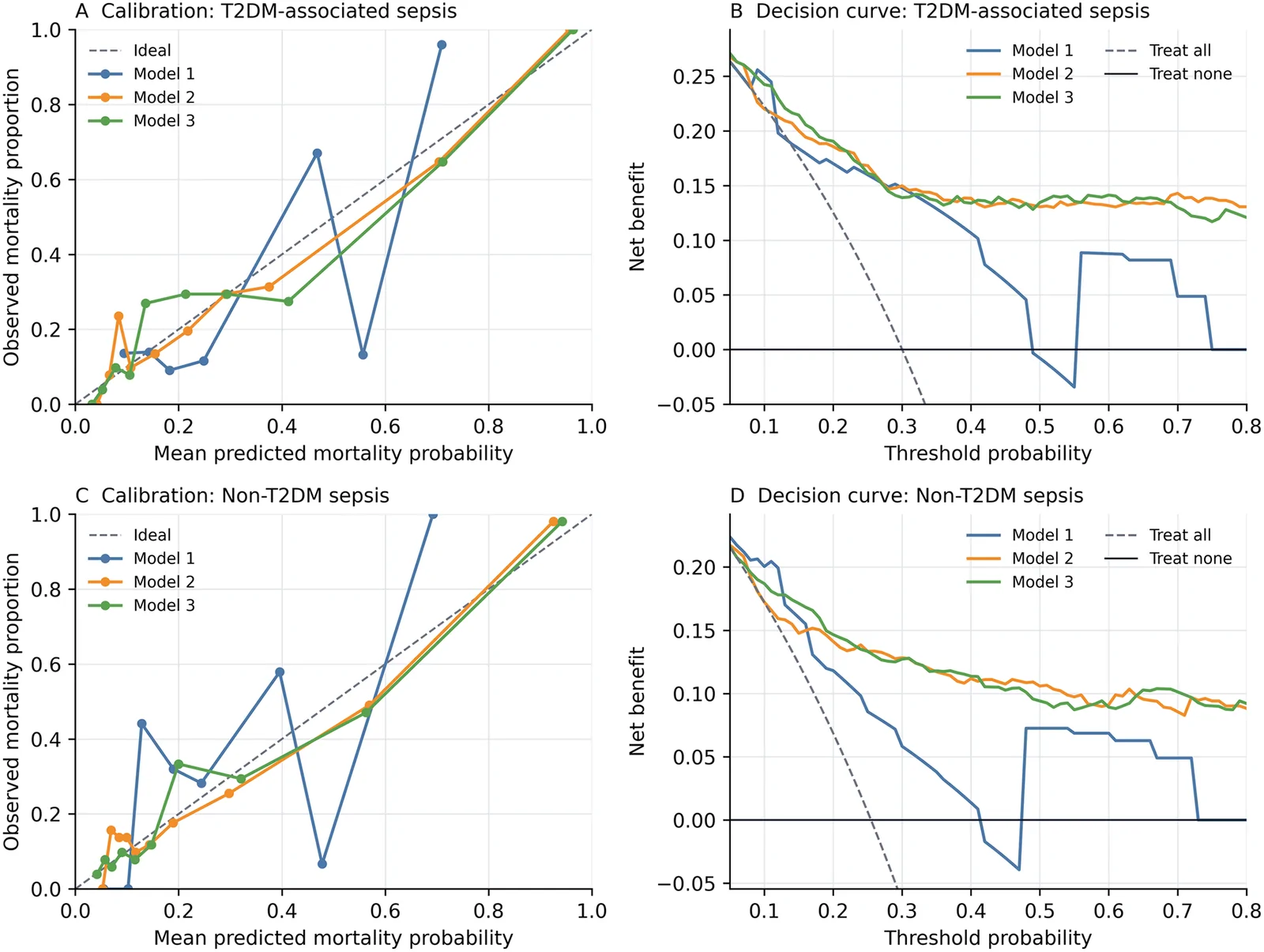
Apparent grouped calibration plots and empirical decision curve analyses for the two cohorts. (A) Calibration in the T2DM cohort; (B) decision curve analysis in the T2DM cohort; (C) calibration in the non-T2DM cohort; and (D) decision curve analysis in the non-T2DM cohort. The decision curves compare Models 1–3 with treat-all and treat-none strategies over threshold probabilities from 0.05 to 0.80. Model 3 had the highest apparent net benefit across approximately 0.12–0.21 in the T2DM cohort and 0.13–0.23 in the non-T2DM cohort, with additional non-continuous regions at higher thresholds. Optimism-corrected calibration intercepts and slopes and apparent Hosmer-Lemeshow *P* values are reported in Table 7. These development-sample findings are exploratory and do not define validated deployment thresholds or establish clinical utility.

Model 3 was designed as a 72-h reassessment model. In the T2DM cohort, ΔSOFA was 2.00 [0.00, 5.00] in non-survivors and −1.00 [−2.00, 1.00] in survivors, indicating persistent early deterioration in organ dysfunction among non-survivors. The overall ΔSOFA distribution was 0.00 [−2.00, 3.00], consistent with the subgroup pattern and outcome direction.

Model 3a produced an AUC of 0.854 (95% CI, 0.819–0.890) in the T2DM cohort and 0.842 (0.801–0.883) in the non-T2DM cohort. The apparent AUC differences favoring Model 3 were small (0.007 and 0.006), with paired DeLong *P* values of 0.001 and 0.315, respectively. Apparent Brier scores for Models 3/3a were 0.123/0.122 in T2DM and 0.114/0.116 in non-T2DM. The nested likelihood ratio test for the additional free coefficient in Model 3 yielded *P* = 0.540 and *P* = 0.027, respectively (Table 9). The likelihood ratio and DeLong tests address different statistical hypotheses; the latter should not be treated as a definitive test of incremental value for these fitted nested models. These findings do not establish generalizable superiority of either parameterization.

**Table 9:** Sensitivity analysis replacing day-1 SOFA plus ΔSOFA with day-3 SOFA.

Cohort	Model	Included variables	AUC (95% CI)	Brier	HL P	DeLong *P* vs. Model 3
T2DM-associated sepsis	Model 3	day-1 SOFA+ΔSOFA+Th1/Th2+IL-6	0.861 (0.827–0.895)	0.123	0.013	Reference
T2DM-associated sepsis	Model 3a	day-3 SOFA+Th1/Th2+IL-6	0.854 (0.819–0.890)	0.122	0.087	0.001
Non-T2DM sepsis	Model 3	day-1 SOFA+ΔSOFA+Th1/Th2+IL-6	0.848 (0.806–0.890)	0.114	0.227	Reference
Non-T2DM sepsis	Model 3a	day-3 SOFA+Th1/Th2+IL-6	0.842 (0.801–0.883)	0.116	0.216	0.315

All values describe apparent performance. Model 3 is the prespecified 72-h reassessment model; Model 3a uses day-3 SOFA. HL indicates the Hosmer-Lemeshow test. DeLong comparisons are paired within cohort and are descriptive for these fitted nested models. The one-degree-of-freedom likelihood-ratio test of equal day-1 SOFA and ΔSOFA coefficients in Model 3 (the Model 3a constraint) gave *P* = 0.540 in T2DM and *P* = 0.027 in non-T2DM. Neither a non-significant difference nor similar AUCs establishes equivalence.

## Discussion

This study shows that patients with sepsis and T2DM are not defined only by a numerically higher crude mortality rate. Crude in-hospital mortality was 30.0% in the T2DM cohort and 25.5% in the non-T2DM sepsis cohort, but this difference was not statistically significant. More relevant clinically, patients with T2DM had higher blood glucose, HbA1c, IL-6, IL-10, lactate, renal dysfunction indices, the NLR, and APACHE II scores, consistent with a heavier immunometabolic and inflammatory burden.

Previous studies have reported mixed associations between diabetes and mortality in sepsis (13, 14). Rather than exerting a uniform binary effect, T2DM may alter inflammatory responses, metabolic stress, endothelial function, and susceptibility to organ injury. In our *post hoc* pooled analysis, the adjusted diabetes status coefficient was negative but did not reach conventional statistical significance (*P* = 0.096), and the global predictor-by-diabetes interaction test was also non-significant. The cohort-specific models should therefore be interpreted as exploratory descriptions of two immunometabolic settings, not as evidence that separate models are required for clinical use. External validation should compare pooled and cohort-specific strategies directly.

From a mechanistic standpoint, hyperglycemia, lipotoxicity, and insulin resistance can impair immune function through oxidative stress, the AGE–RAGE axis, NF-κB signaling, mitochondrial dysfunction, and endothelial injury (15, 16). Recent studies also indicate that T2DM is accompanied by changes in immune cell composition, metabolic pathways, and T-cell activation programs (17, 18). In the present cohort, higher concentrations of IL-6, IL-10, lactate, and renal dysfunction markers in patients with T2DM were consistent with this pattern of immunometabolic reprogramming.

The Th1/Th2 ratio reflects the orientation of T-helper-cell polarization. Sepsis-associated immune dysfunction is not a uniform proinflammatory or immunosuppressive state, but a shifting imbalance involving innate immunity, T-cell exhaustion or activation, endothelial injury, coagulation abnormalities, and metabolic reprogramming (19, 20). In this study, non-survivors in both cohorts had higher Th1/Th2 ratios, and the Th1/Th2 ratio remained independently associated with mortality in the T2DM cohort. This is consistent with evidence linking T-cell subsets and lymphocyte immune profiles to sepsis severity and clinical outcome (21, 22). However, an elevated Th1/Th2 ratio may result from increased Th1 cells, reduced Th2 cells, or both; future work should include absolute cell counts and serial immune monitoring.

IL-6 links systemic inflammation with metabolic stress. Both baseline IL-6 concentrations and changes over time have been associated with sepsis mortality, and IL-6 has been examined repeatedly as a clinically useful sepsis biomarker (23, 24). In our cohort, IL-6 and IL-10 were both elevated among non-survivors, suggesting that fatal outcomes may reflect concurrent inflammatory activation and compensatory anti-inflammatory feedback rather than a purely unidirectional inflammatory response. Although the NLR and lactate were not included in the main model, their univariable signals were substantial, confirming that cellular inflammatory imbalance and tissue hypoperfusion remain important contextual indicators (25, 26).

SOFA data quality control was an important methodological step. The theoretical SOFA range is 0–24 points, and all SOFA scores in this study were checked against this range and reviewed for organ subscore consistency. In the T2DM non-survivor group, day-1 SOFA was 15.00 [12.00, 18.00] and day-3 SOFA was 16.00 [13.00, 19.00], indicating severe organ dysfunction without implausible clustering near the upper limit. Prior evidence supports the prognostic value of SOFA, lactate, PCT, qSOFA, and SOFA-derived indicators in sepsis (7, 27). The development of SOFA-2 further highlights the need to reassess organ dysfunction scores in contemporary ICU populations (28).

Age is inexpensive, immediately available, and prognostically important. In this study, however, age alone discriminated mortality substantially less well than Model 3. Adding age modestly improved Brier scores and increased the AUC by approximately 0.009 in the T2DM cohort, but the AUC increase was not statistically significant in either cohort. The age coefficient itself remained significant in both age-augmented models. We therefore do not regard age as uninformative. Rather than redefining the prespecified primary Model 3 after observing the data, we retained Model 3 plus age as a transparent secondary benchmark. Age should remain a candidate predictor in future externally validated models, where its value should be judged by calibration, overall accuracy, clinical utility, and discrimination rather than by AUC alone.

This development analysis provided internal support for the staged immunodynamic hypothesis. Model 3 achieved apparent/corrected AUCs of 0.861/0.858 in the T2DM cohort and 0.848/0.841 in the non-T2DM cohort. Figure 3 compares apparent and optimism-corrected AUC point estimates. These displays do not quantify sampling uncertainty. Corrected calibration intercepts were near 0, and slopes were near 1, but the apparent Hosmer–Lemeshow goodness-of-fit test for the T2DM Model 3 was significant (*P* = 0.013). This pattern indicates little average calibration offset or linear shrinkage while allowing for groupwise or local miscalibration; we therefore do not characterize the model as well calibrated. Recalibration and independent validation are required before clinical implementation. Decision curve analysis showed higher apparent net benefit for Model 3 across selected threshold ranges, but those ranges came from the development dataset and are not validated decision cutoffs. Whether the added discrimination justifies the cost of biomarker measurement and produces measurable clinical benefit will depend on net benefit at clinically chosen thresholds and must be tested in prospective impact and cost-effectiveness studies. Current guidance emphasizes discrimination, calibration, validation, and clinical utility rather than AUC alone (8, 29, 30).

Day-3 SOFA integrates substantial prognostic information. Expressing the 72-h assessment using separate day-1 SOFA and ΔSOFA terms distinguishes baseline organ dysfunction from subsequent change, while adding one free coefficient relative to Model 3a. The small apparent AUC differences and the differing likelihood ratio and DeLong results do not demonstrate that this additional complexity yields a consistently better model. Retaining the prespecified Model 3 allows a separate description of baseline dysfunction and trajectory, but clinical interpretability should not be equated with proven predictive or clinical superiority. Both parameterizations require external validation.

The intended clinical use is a structured risk reassessment at 72 h among patients with similar SOFA trajectories. The model should not be applied when any required predictor is missing, measured outside the prespecified assessment window, or analytically unreliable. Implementation would require clinician review of data quality and automated range checks, including confirmation that SOFA scores fall within the 0–24-point theoretical range. The intended users are ICU clinicians trained to interpret the output in conjunction with the full clinical assessment. Consider two hypothetical patients in the T2DM cohort, each with a day-1 SOFA score of 7 and a ΔSOFA of 0. Using the reported Model 3 equation, a patient with an IL-6 concentration of 100 pg/mL and a Th1/Th2 ratio of 0.5 has a development-sample predicted mortality probability of approximately 4.6%. A second patient with an IL-6 concentration of 700 pg/mL and a Th1/Th2 ratio of 1.4 has a predicted probability of approximately 21.4%. These calculations illustrate how the biomarker terms may rerank patients despite identical SOFA trajectories; they are examples, not validated cutoffs. The higher-ranked patient could be prioritized for senior or multidisciplinary review of source control, diagnostic imaging, antimicrobial adequacy, perfusion, organ support requirements, and monitoring intensity. The model does not mandate a specific intervention, and discussions of prognosis or goals of care remain matters of clinical judgment. Prospective impact studies are needed before these uses can be recommended.

Lymphocyte and lactate trajectories may provide additional support for dynamic risk assessment, as both have been associated with sepsis prognosis in recent studies (31, 32). Single-timepoint measurements rarely capture the full course of sepsis pathophysiology, whereas serial changes more closely mirror bedside decision-making in the ICU.

Several limitations warrant emphasis. Because day 1 was anchored to ICU admission, pre-ICU ward treatment and variable hospital-to-ICU transfer intervals could not be fully accounted for; residual lead-time bias may therefore remain. First, the single-center retrospective design may have introduced selection bias, treatment confounding, and timing bias. Although the ICU glycemic target was standardized, individual insulin exposure, glucose variability, hypoglycemia, and target adherence were unavailable; residual glycemic treatment confounding remains. Medical Records Department staff retrieved the electronic data, and three ICU physicians reviewed the dataset. Although the core model variables passed internal consistency checks, the audit trail did not record field-level abstractor identity or independent duplicate abstraction, and formal interrater agreement was unavailable. Second, the 72-h landmark required an ICU stay of at least 72 h and paired day-1/day-3 SOFA scores. The day-level ICU-stay variable in the analysis export cannot independently confirm exact elapsed hours; the 72-h criterion reflects the reported source-record eligibility rule. This requirement excluded early deaths and rapid discharges, introduced survivor and selection bias, and prevents use of the model during the hyperacute phase. Because Models 1–3 were compared in the same landmark cohort, all performance estimates—including those for the baseline-only models—are conditional on survival and evaluability at day 3. SOFA is bounded, and ΔSOFA mathematically includes the baseline score; floor and ceiling effects, regression to the mean, and mathematical coupling may therefore influence the estimated ΔSOFA association. The absence of reliable times to final hospital death and live discharge also prevented valid time-to-event or competing-risk sensitivity analyses. Third, Th1/Th2 and cytokines were measured mainly once, and separate Th1 and Th2 percentages were not retained in the analysis export. Because absolute CD3+CD4+, Th1, and Th2 counts were unavailable, the percentage-based Th1/Th2 ratio cannot distinguish relative Th1 expansion, relative Th2 reduction, or changes occurring in the setting of generalized CD4+ T-cell lymphopenia. Between-center harmonization was not assessed. Fourth, we used complete cases from the 72-h landmark cohort. Although no analyzed variable was missing among included patients, variable-level partial data for excluded patients could not be reconstructed. We therefore could not evaluate the missing-completely-at-random assumption for those exclusions or perform a multiple-imputation sensitivity analysis. Fifth, causative organisms, community- vs. hospital-acquired infection, HIV status, and detailed chronic comorbidity data were unavailable, limiting assessment of generalizability. The study was not designed to evaluate health inequalities across sociodemographic groups; race and ethnicity, socioeconomic status, and related structural determinants were unavailable in the retrospective dataset. Sixth, the age and pooled analyses were *post hoc* and require confirmation in independent data. Seventh, validation was limited to internal bootstrap resampling of fixed model specifications; it does not account for any undocumented data-dependent decisions preceding model specification. Confidence intervals for the optimism-corrected point estimates were not estimated. Neither bootstrap validation nor a random split can establish temporal or geographic transportability. The calibration and decision-curve results were derived from the development sample, and neither prospective clinical impact nor cost-effectiveness was evaluated. A later cohort from the same center and multicenter external cohorts are needed to evaluate discrimination, calibration, recalibration requirements, clinical utility, coefficients, intercepts, and decision thresholds with adequate precision (33).

## Conclusion

Patients with sepsis and T2DM manifested a greater inflammatory, glycometabolic, renal, and physiologic severity burden than patients without T2DM. Within the 72-h landmark cohort, a model combining day-1 SOFA, ΔSOFA, the Th1/Th2 ratio, and IL-6 demonstrated superior apparent and bootstrap-corrected discrimination than SOFA alone. These performance estimates apply only to patients who survived and remained evaluable at day 3. Age was associated with mortality, but it added little discrimination after SOFA trajectory and immune-inflammatory variables were included; the age-augmented model is therefore reported transparently as a *post hoc* benchmark. In the pooled age-adjusted model, the diabetes status coefficient was negative but did not reach conventional statistical significance (*P* = 0.096), and the global predictor-by-diabetes interaction test was also non-significant. These models are investigational aids for prioritizing a structured 72-h reassessment, not replacements for SOFA or prescriptive rules. Temporal and multicenter external validation, including recalibration and prospective assessment of clinical utility, is required before implementation.

## Data Availability

The datasets analyzed during this study are not publicly available because they contain patient-level clinical information. De-identified data may be available from the corresponding authors upon reasonable request and subject to institutional approval. Aggregate results and analysis and verification code are provided in the Supplementary Material. These supplementary files contain no patient-level clinical records.
